# Developmental stage-dependent regulation of spine formation by calcium-calmodulin-dependent protein kinase IIα and Rap1

**DOI:** 10.1038/s41598-017-13728-y

**Published:** 2017-10-17

**Authors:** Solveigh Cornelia Koeberle, Shinji Tanaka, Toshihiko Kuriu, Hirohide Iwasaki, Andreas Koeberle, Alexander Schulz, Dario-Lucas Helbing, Yoko Yamagata, Helen Morrison, Shigeo Okabe

**Affiliations:** 1 Department of Cellular Neurobiology, Graduate School of Medicine, University of Tokyo, Tokyo, Japan; 2 Institute of Age Research, Fritz Lipmann Institute, Jena, Germany; 3 Department of Biochemistry and Molecular Biology, Faculty of Medicine, The University of Tokyo, Tokyo, Japan; 4 Department of Pharmaceutical/Medicinal Chemistry, Institute of Pharmacy, Friedrich-Schiller-University, Jena, Germany; 5 Division of Neural Signaling, National Institute for Physiological Sciences, Okazaki, 444-8787 Japan; 6 SOKENDAI (The Graduate University for Advanced Studies), Okazaki, 444-8787 Japan; 7 Department of Neurophysiology, Kagawa School of Pharmaceutical Sciences, Tokushima Bunri University, Kagawa, 769-2193 Japan; 8 CREST, JST, Japan

## Abstract

The roles of calcium-calmodulin-dependent protein kinase II-alpha (CaMKIIα) in the expression of long-term synaptic plasticity in the adult brain have been extensively studied. However, how increased CaMKIIα activity controls the maturation of neuronal circuits remains incompletely understood. Herein, we show that pyramidal neurons without CaMKIIα activity upregulate the rate of spine addition, resulting in elevated spine density. Genetic elimination of CaMKIIα activity specifically eliminated the observed maturation-dependent suppression of spine formation. Enhanced spine formation was associated with the stabilization of actin in the spine and could be reversed by increasing the activity of the small GTPase Rap1. CaMKIIα activity was critical in the phosphorylation of synaptic Ras GTPase-activating protein (synGAP), the dispersion of synGAP from postsynaptic sites, and the activation of postsynaptic Rap1. CaMKIIα is already known to be essential in learning and memory, but our findings suggest that CaMKIIα plays an important activity-dependent role in restricting spine density during postnatal development.

## Introduction

Calcium-calmodulin-dependent protein kinase II (CaMKII) is a serine/threonine kinase that is highly expressed in the brain^1^. CaMKIIα and β are the major isoforms expressed in the nervous system, and their expression is associated with distinct developmental profiles. Because α and β isoforms have unique biochemical properties, CaMKIIα and β have been proposed to play isoform-specific roles in specific developmental stages^2,3^. Synaptic accumulation of CaMKIIα in the late postnatal stage of forebrain development promotes the hypothesis that CaMKIIα selectively controls synaptic efficacy in the adult brain. However, no precise evaluation of neural circuit development based on imaging of individual synapses and dendritic spines has been conducted.

The number of synapses in the rodent neocortex and hippocampus increases dramatically during the early postnatal stage^4,5^. Recent *in vivo* imaging of cortical pyramidal neurons revealed that extensive spine remodelling occurs in the early postnatal period^6^. The total spine number peaks near postnatal week 3 and declines slowly thereafter. This developmental profile is regulated by the balance between spine addition and spine elimination^6–8^. Previous studies produced evidential support for the roles of plasticity-associated molecules in spine remodelling in the developing cortex^9^. Of these plasticity-related signalling mechanisms, NMDA receptor-dependent signalling has been shown to be essential in long-term changes in synaptic functions and structures^10^. Genetic manipulation of NMDA receptor functions in the cortex dramatically affects the connectivity of thalamic afferents to cortical pyramidal neurons^11^. CaMKIIα plays an important role in the phosphorylation of multiple target proteins, such as synaptic Ras GTPase-activating protein (synGAP)^1,12,13^, in the signalling cascade downstream of NMDA receptor activation. CaMKIIα is involved in both synaptic efficacy and spine head enlargement during LTP^14,15^. Moreover, synGAP, a protein expressed in the postsynaptic density, may act downstream of CaMKIIα to regulate small GTPases Ras and Rap during LTP^16–18^. We previously generated knock-in mice lacking kinase activity but with physiological expression of a mutated CaMKIIα protein (K42R)^19^. This mouse model should be useful in detecting the regulatory functions of CaMKIIα-dependent signalling pathways in the development and maturation of synaptic connectivity in the mouse forebrain.

The combination of the late onset of CaMKIIα accumulation, the important role of CaMKIIα in synaptic plasticity, and the involvement of NMDA receptor-dependent signalling in proper cortical neuron wiring led us to perform detailed structural analyses of dendritic spine development in hippocampal slice cultures from CaMKIIα (K42R) knock-in mice (CaMKIIα KI mice). We found that pyramidal neurons without CaMKIIα activity upregulated the rate of spine addition, which resulted in elevated spine density. This enhancement of spine formation was associated with the stabilization of actin in the spines and a reduction in the activity of the small GTPase Rap1. Our findings suggest that gradual increases in CaMKIIα activity in the postnatal forebrain may be effective in suppressing the rapid increase in spine synapse density via the activation of Rap1 signalling.

## Results

### Enhancement of spine growth in CaMKIIα KI hippocampal slices

**Figure 1:**
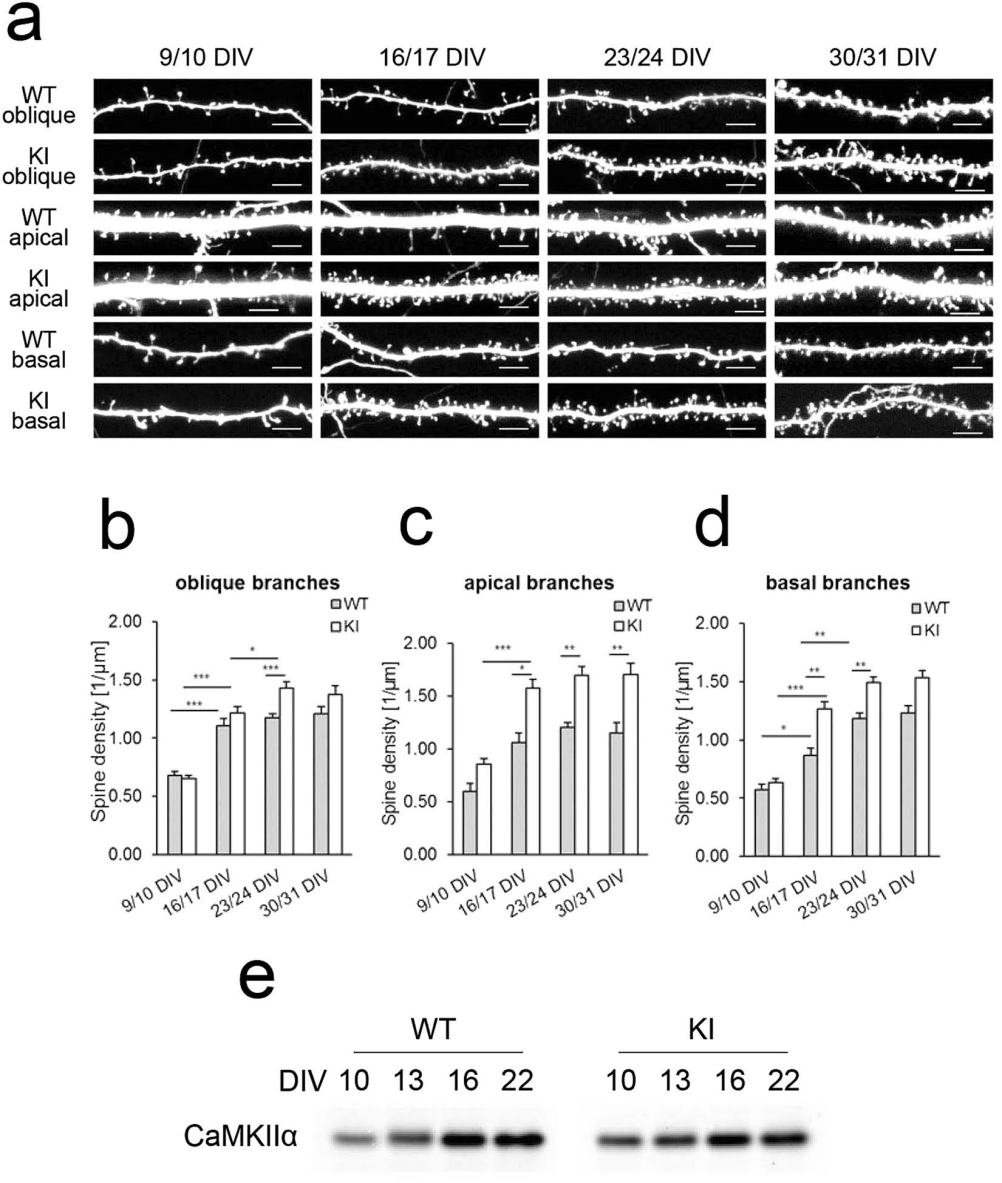
Spine densities of wild-type and CaMKIIα KI hippocampal neurons maintained in slice cultures. (**a**) Representative images of oblique, apical, and basal dendrites at different developmental stages. Scale bars, 5 µm. (**b**–**d**) Quantitative analysis of spine density in hippocampal pyramidal neurons maintained in slice cultures of wild-type and CaMKIIα KI mice at 9/10 DIV, 16/17 DIV, 23/24 DIV and 30/31 DIV. Spine density was measured from images of oblique (**b**), apical (**c**), and basal (**d**) branches of dendrites, collected from 7–22 experiments. Data are presented as the mean ± SEM, n = 7–22 cells, *p < 0.05, **p < 0.01 or ***p < 0.001, two-way ANOVA followed by Tukey’s post-hoc test. (**e**) The protein expression of CaMKIIα at 10, 13, 16 and 22 DIV in slices from KI and wild-type mice. CaMKIIα protein expression was low at 10 DIV in both KI and wild-type mice. A large increase in protein expression was observed from 10 to 16 DIV. Full-size images of the gels and blots are shown in Supplementary Fig. 1.

Spine density increases until the third postnatal week in the rodent hippocampus and remains relatively constant thereafter^20–22^. To determine whether this developmental profile was preserved in a hippocampal slice culture, we expressed GFP in a small subset of neurons, performed high-resolution confocal imaging of multiple dendritic segments from CA1 pyramidal-shaped neurons, and measured spine density (Fig. 1a). We confirmed similar profiles of spine density increases in these slice cultures. Specifically, spine densities in oblique, apical, and basal dendrites increased between 9 days *in vitro* (DIV) and 17 DIV, and plateaued after 17 DIV (Fig. 1b–d). Hippocampal slice cultures prepared from CaMKIIα KI and wild-type mice showed no differences in the survival of pyramidal neurons or the overall organization of the pyramidal cell layers. There were no significant differences in spine density, as measured at multiple dendritic subcompartments, in the early stage of maturation (9 DIV). However, CaMKIIα KI neurons continued to exhibit increased spine density even after 17 DIV, resulting in significant differences between the genotypes in all dendrite subcompartments (oblique, apical, and basal) at 24 DIV (Fig. 1b–d). To test whether the timing of arrest in spine increases correlated with the upregulation of CaMKIIα expression, we quantitated the level of CaMKIIα protein by western blot analysis of proteins extracted from hippocampal slice cultures (Fig. 1e and Supplementary Fig. 1). The expression profiles confirmed that CaMKIIα is upregulated from P10 to P16 and that its level stabilizes thereafter, irrespective of the genotype. Consistent with the result of a previous report^19^, immunoblot analysis showed that phospho-CaMKIIα levels were decreased in CaMKIIα KI slices. However, the expression profiles of phospho-CaMKIIα levels showed trends similar to those of CaMKIIα in both genotypes (Supplementary Figure 1). This expression profile of CaMKIIα was consistent with its role in the suppression of increased spine density in hippocampal slices after 17 DIV.

### Dynamics and activity-dependent regulation of spines in CaMKIIα KI slices

**Figure 2:**
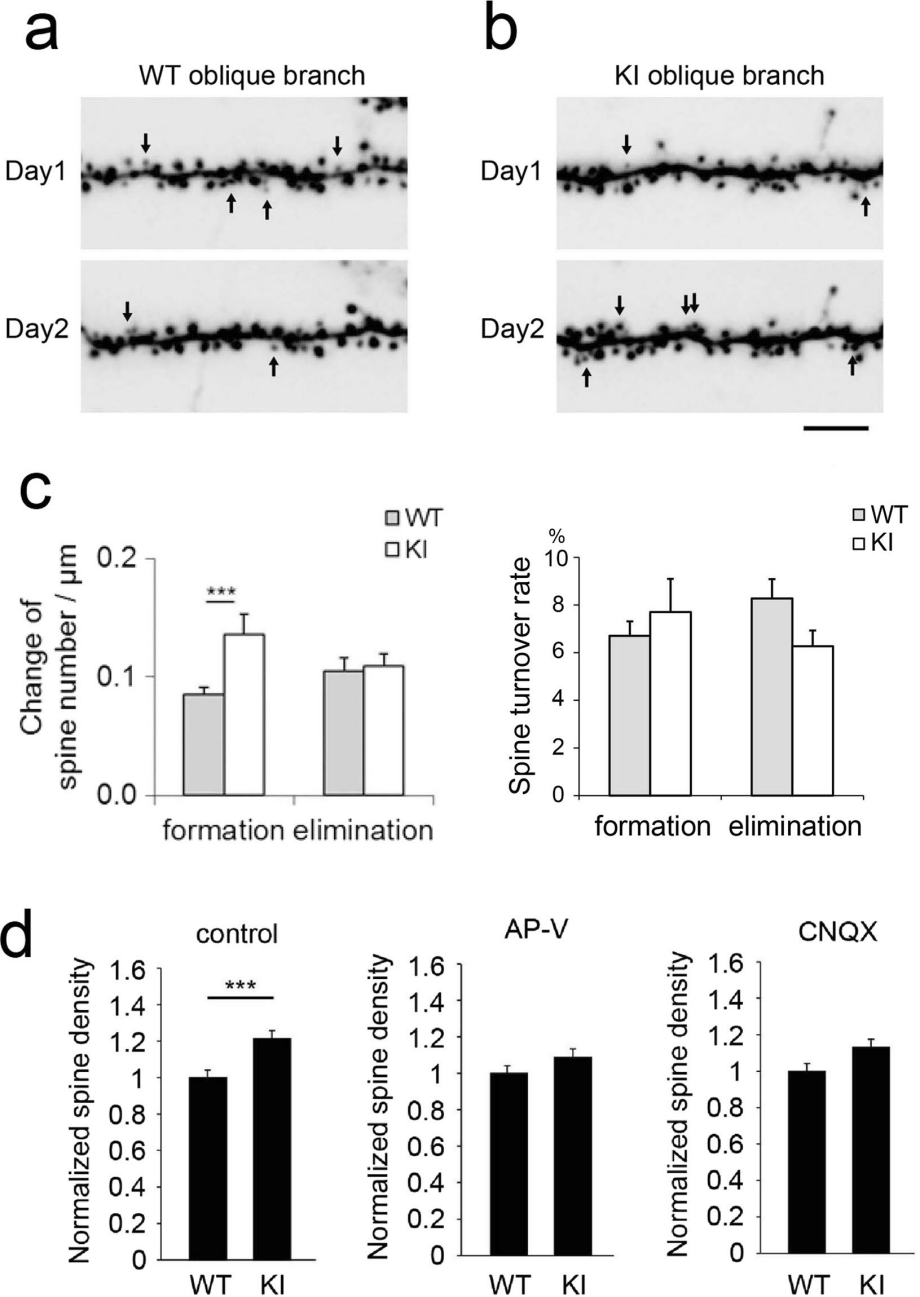
Spine formation and elimination in CA1 pyramidal neurons in CaMKIIα KI and wild-type hippocampal slices. (**a** and **b**) Spines formed (arrows at Day2) and eliminated (arrows at Day1) in the oblique branches of CA1 pyramidal neurons in mature slices (19–23 DIV), as detected by time-lapse imaging of GFP-expressing cells over 24 h. The dendritic segments of wild-type (**a**) and KI (**b**) slices are presented. Scale bars, 5 µm. (**c**) Quantitative analysis of spine formation and elimination in wild-type and KI mice. Data are presented as the mean ± SEM, n = 5 cells from 2 independent cultures for wild-type mice, n = 5 cells from 3 independent cultures for KI mice, *p < 0.05, t-test. (**d**) Inhibiting AMPA receptors with CNQX (20 µM), and NMDA receptors with AP-V (50 µM) for 4 days (from 19 to 23 DIV) reduced the spine density of KI neurons to wild-type neuron levels. Control spine densities were based on the same set of the data shown in Fig. 1. Data are presented as the mean ± SEM, n = 21 and 37 cells from 10 and 11 independent cultures of WT and KI control, n = 10 and 9 cells from 3 independent cultures of WT and KI APV treat ment, n = 13 and 23 cells from 4 independent cultures of WT and KI CNQX treat ment, ***p < 0.001, t-test.

To investigate whether the increased spine density in CaMKIIα KI neurons resulted from increased spine addition or reduced spine elimination, we maintained slice cultures for approximately three weeks *in vitro* (19–22 DIV) and performed time-lapse imaging with an interval of 24 h. We then evaluated the turnover of dendritic spines on oblique dendritic branches (Fig. 2a,b). The difference in spine density between CaMKIIα KI and wild-type mice was significant at this developmental stage. The rate of spine addition was slightly lower than the rate of spine elimination in wild-type mice (Fig. 2c), a result that explains the stagnation of spine density after 17 DIV. Spine addition was significantly enhanced in CaMKIIα KI mice; however, spine elimination did not differ between the two genotypes. This specific enhancement of spine addition may be the mechanism underlying the observed sustained increase in spine density in CaMKIIα KI neurons.

Because ionotropic glutamate receptors are major upstream signalling molecules involved in CaMKII activation in neurons, we next tested whether the pharmacological blockade of either AMPA or NMDA receptors in slice cultures is involved in CaMKII-dependent spine regulation. Application of the AMPA receptor antagonist CNQX or the NMDA receptor antagonist AP-V for 4 days (19 to 23 DIV) effectively eliminated the difference in spine density between CaMKIIα KI and wild-type neurons (Fig. 2d). This observation is consistent with the idea that CaMKIIα activity negatively modulates spine formation via a pathway related to glutamate receptor signalling.

### CaMKII-dependent regulation of increased spine number and spine maintenance in dissociated neurons

**Figure 3:**
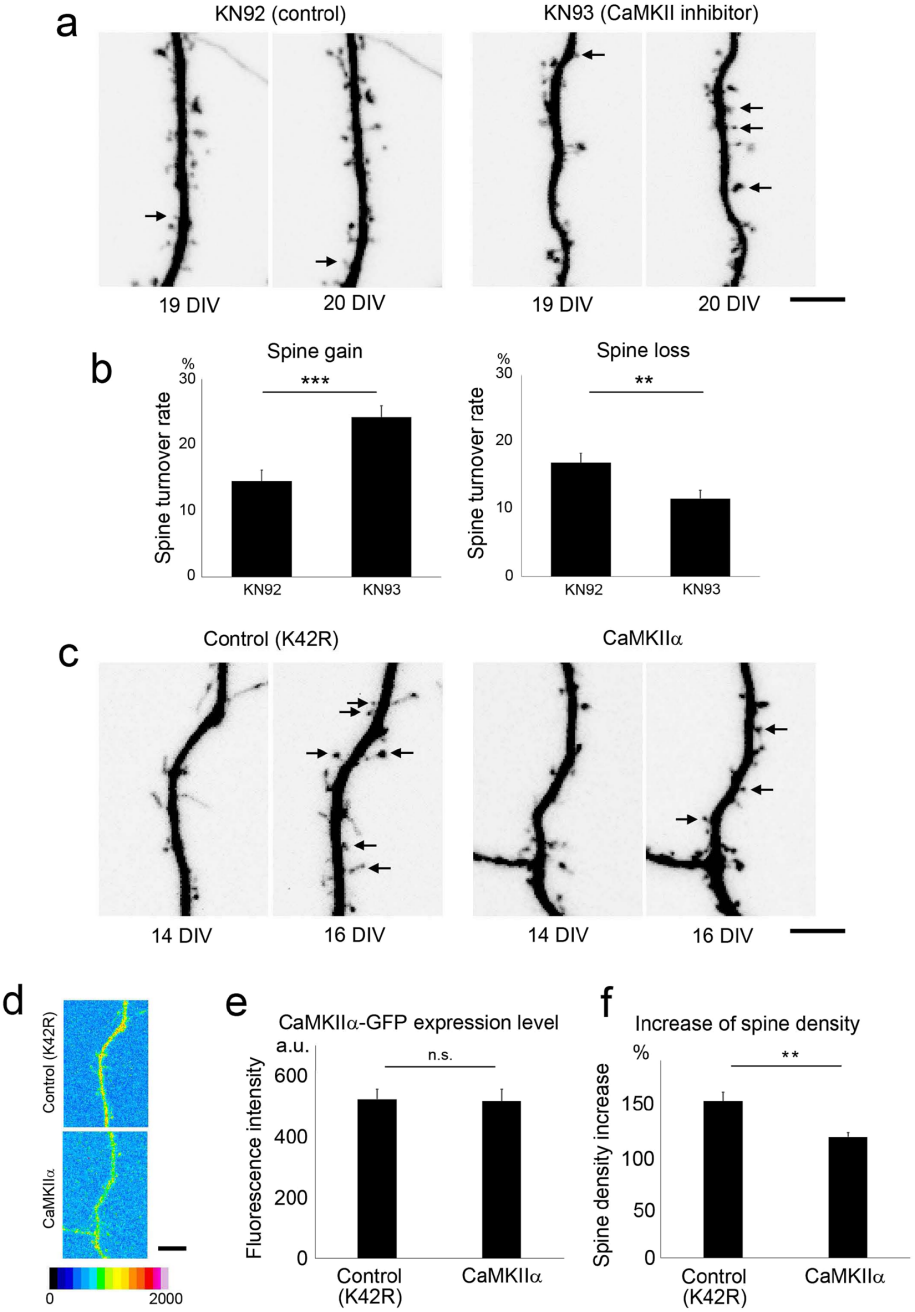
Regulation of spine dynamics by CaMKII in dissociated neurons. (**a**) Time-lapse imaging of spines in dissociated hippocampal neurons expressing RFP and treated with the CaMKII inhibitor KN93 or its inactive analogue KN92. Gain and loss of spines are marked by arrows. Bar, 5 µm. (**b**) Enhancement of the spine turnover rate in neurons treated with KN93. Data are presented as the mean ± SEM, (neurons at 19–20 DIV, n = 12 cells from 3 independent cultures), ***p < 0.001, **p < 0.01, t-test. (**c**) Time-lapse imaging of spines in dissociated hippocampal neurons expressing RFP along with GFP-tagged wild-type CaMKII or the kinase-dead mutant of CaMKII (K42R). Newly formed spines are marked by arrows. Bar, 5 µm. (**d**) Pseudocolour images of neurons expressing GFP-tagged wild-type CaMKIIα or the K42R mutant at similar levels. Bar, 5 µm. (**e**) Quantification of the expression level of GFP-tagged wild-type CaMKIIα or the K42R mutant. Data are presented as the mean ± SEM (neurons at 14–16 DIV, n = 11 cells from 3 independent cultures for wild-type CaMKIIa, n = 20 cells from 3 independent cultures for K42R CaMKIIα), n.s. p > 0.05, t-test. (**f**) Quantification of the increase in spine density from 14 to 16 DIV in neurons expressing GFP-tagged wild-type CaMKIIα or the K42R mutant. Data are presented as the mean ± SEM, (neurons at 14–16 DIV, n = 11 cells from 3 independent cultures for wild-type CaMKIIa, n = 20 cells from 3 independent cultures for K42R CaMKIIα), **p < 0.01, t-test.

Analyses of hippocampal slice cultures provided strong evidence indicating that spine density regulation is CaMKIIα dependent. However, it could be argued that the spine phenotype is a result of prior effects of CaMKIIα on circuit formation and other non-synaptic functions. To evaluate this possibility, we next tested whether the acute blockade of CaMKII activity by specific inhibitors in dissociated hippocampal neurons can induce similar changes in spines (Fig. 3a). Previous studies have reported that CaMKIIα is upregulated in dissociated neurons maintained in culture for more than 2 weeks *in vitro* ^2^. Therefore, we applied the CaMKII blocker KN93 to neurons and performed time-lapse imaging at 19 to 20 DIV. KN93 treatment induced the upregulation of spine turnover. The trends in spine gain and loss observed in these cultures were similar to those observed in slice cultures (Fig. 3b). This result supports the idea that the effect of CaMKIIα suppression on late-stage spine turnover is not indirectly caused by developmental alterations in circuit formation. To test whether CaMKII activity can bidirectionally regulate spine development, we overexpressed either wild-type or kinase-dead K42R CaMKIIα in dissociated neurons at earlier time points (9–16 DIV), when CaMKIIα expression is still moderate^2^. We then monitored the increase in spine density from 14 to 16 DIV (Fig. 3c–f). For these experiments, we used K42R CaMKIIα as a control to compare the effects of the presence or absence of the enzymatic activity of exogenous CaMKIIα on spine density while keeping the level of overexpressed protein comparable (Fig. 3d). As expected, overexpression of wild-type CaMKIIα specifically suppressed the increase in spine density; however, K42R CaMKIIα failed to suppress this increase (Fig. 3f). We did not attempt to evaluate the spine phenotype of CaMKIIα KI neurons in dissociated cultures because spine density and maturation-dependent regulation of spine turnover are sensitive to initial plating conditions. Nevertheless, the analyses of slices taken from CaMKIIα KI mice and dissociated neurons that were manipulated pharmacologically or with gene transfection revealed that spine turnover is regulated in a CaMKIIα-dependent manner in both slice and dissociated culture conditions.

### Downregulation of the small GTPase Rap1 by CaMKII inactivation

**Figure 4:**
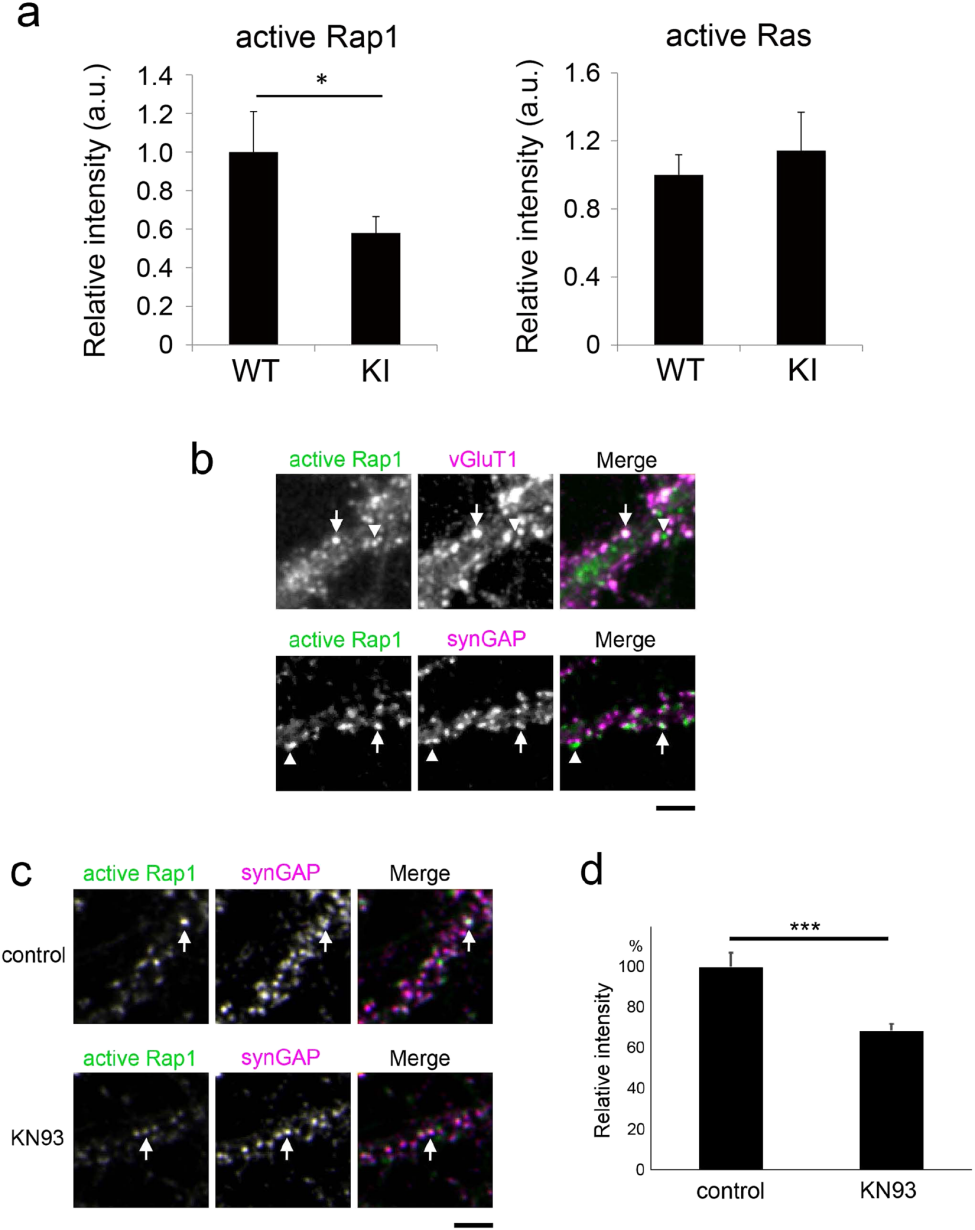
CaMKII-dependent regulation of Rap1 activity. (**a**) Quantification of active Rap1 and active Ras immunoreactivity in the dendrites of CaMKIIα KI or wild-type neurons. Data are presented as the mean ± SEM, (neurons at 21–22 DIV, Rap1; n = 42 and 27 cells from 4 independent cultures of wild-type and CaMKIIα KI hippocampi, Ras; n = 32 and 33 cells from 5 independent cultures of wild-type and CaMKIIα KI hippocampi), *p < 0.05, t-test. (**b**) Immunoreactivity of active Rap1 in the dendrites of hippocampal neurons at 22–23 DIV and immunostaining for the presynaptic marker vGluT1 or the postsynaptic marker synGAP. Active Rap1-positive punctae are either colocalized with (arrows) or separated from (arrowheads) the synaptic markers. Bar, 5 µm. (**c**) Immunocytochemistry of KN93-treated neurons exposed to the anti-active Rap1 antibody. The intensity of active-Rap1 punctae at synGAP-positive postsynaptic sites was lower in neurons treated with KN93 (arrows). Bar, 5 µm. (**d**) Quantification of active Rap1 immunoreactivity at synGAP-positive postsynaptic sites indicates that Rap1 activity is negatively regulated by KN93. Data are presented as the mean ± SEM, (neurons at 20–22 DIV, n = 22 cells from 3 independent cultures for both control (KN92) and KN93 treatment), ***p < 0.001, t-test.

Ras and Rap are members of the Ras small GTPases and are involved in activity-dependent changes in synaptic function, spine morphology, and stability^23–25^. Moreover, these proteins are also known to be controlled by CaMKII^13,14^. To test whether Ras small GTPase activity is regulated by CaMKIIα, we quantitated the immunoreactivity of wild-type and CaMKIIα KI neurons in dissociated cultures, using antibodies that selectively recognize the active, GTP-bound conformation of Rap1 and Ras. Rap1 activity levels were specifically reduced in CaMKIIα KI neurons, whereas active Ras levels were not affected (Fig. 4a). Classical pull-down assays confirmed that Rap1-GTP levels were reduced; however, the GTP-bound form of Ras did not show any detectable changes in its levels in hippocampal extracts from CaMKIIα KI mice (Supplementary Fig. 2).

This observation, as well as the observation that spine density is increased in CaMKIIα KI neurons, is consistent with a previous finding indigcating that the activation of Rap reduces spine size or density^24,26^. To further characterize the relationship between Rap1 activation and CaMKII activity in dendrites, we performed high-resolution imaging of synaptic Rap1 activation using the anti-active Rap1 antibody. The active Rap1 antibody recognized punctate structures along dendrites, and double-labelling with synaptic markers revealed that active-Rap1-positive punctae partially overlapped with presynaptic vesicular glutamate transporter 1 (vGluT1) or postsynaptic synGAP immunoreactivity (Fig. 4b). This result suggests that Rap1 is activated in subsets of both presynaptic and postsynaptic components. Because the overlap between punctate structures that were immunopositive for presynaptic and postsynaptic markers was much lower than the overlap between two postsynaptic markers (Supplementary Fig. 3), we could judge whether active Rap1-positive puncta were presynaptic or postsynaptic. Using this criterion, we next tested whether the suppression of CaMKII activity by KN93 affects the level of active Rap1 at postsynaptic sites, as detected by synGAP immunoreactivity (Fig. 4c). We observed that postsynaptic Rap1 activity is down-regulated in KN93-treated hippocampal neurons, suggesting that Rap1 is activated in a CaMKII-dependent manner even in the setting of basal neuronal activity (Fig. 4d).

### The Spatio-temporal dynamics of Rap1 activity are maintained by CaMKII, as determined by fluorescent resonance energy transfer

**Figure 5:**
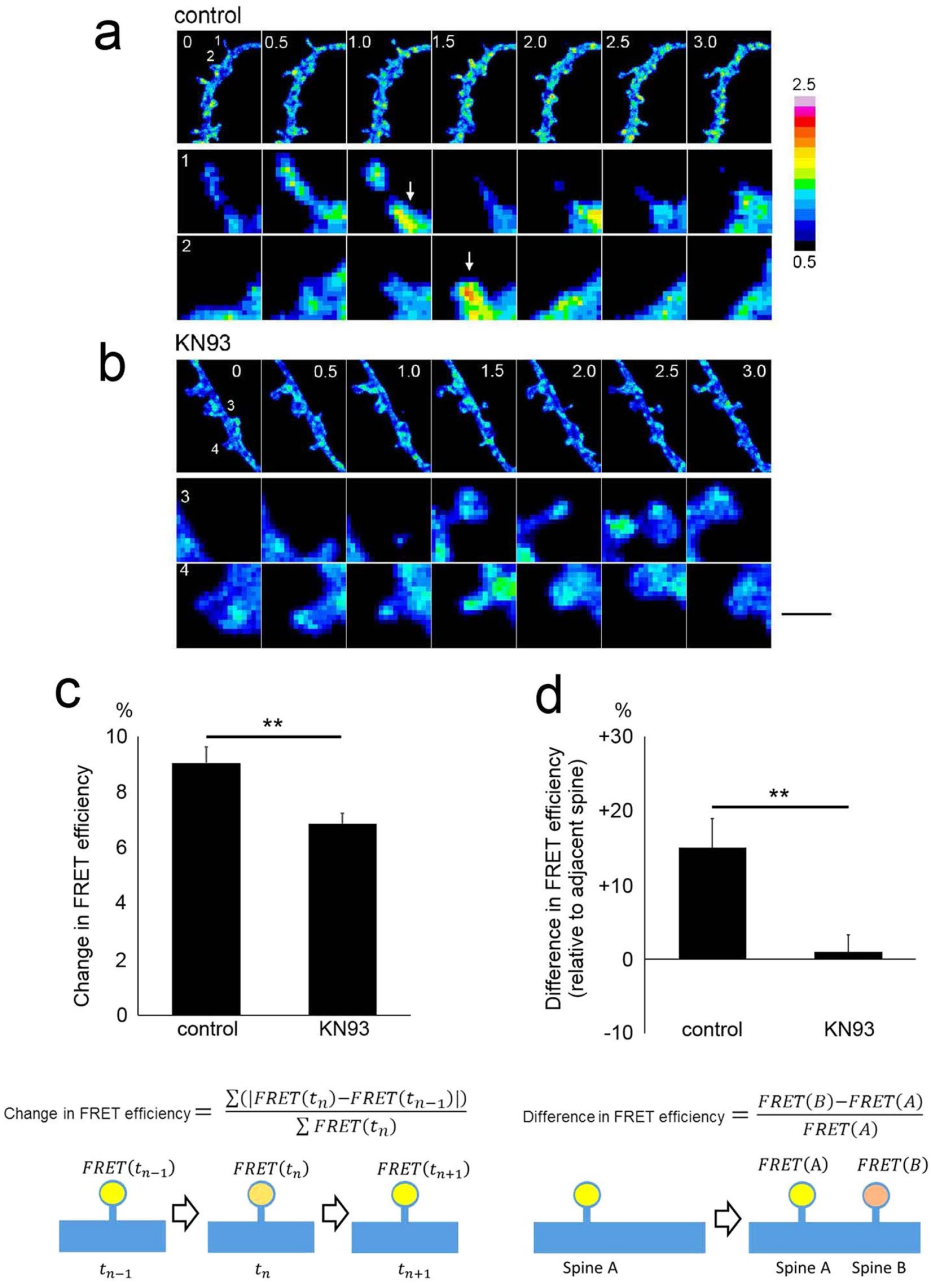
FRET imaging of Rap1 activity in neurons with or without the CaMKII inhibitor. (**a** and **b**) Time-lapse FRET imaging of Rap1 activity in dissociated hippocampal neurons treated with the CaMKII inhibitor (**b**) or its inactive analogue (**a**). Time stamps (hour) are shown in the upper-left-hand corner of the images. Two spines (marked as [1] and [2] for [A] and [3] and [4] for [B]) are shown as enlarged images in the lower rows. The arrows indicate local Rap1 activation. Bar, 5 µm and 1 µm for the lower- and higher-magnification images, respectively. (**c**) Quantification of the changes in FRET efficiency in persistent spines, changes indicative of the suppression of transient Rap1 activation by the inhibition of CaMKII activity. Data are presented as the mean ± SEM, (neurons at 16–21 DIV, n = 24 spines from 3 independent time-lapse sessions for both control [KN92] and KN93), **p < 0.01, t-test. (**d**) Difference in FRET efficiency between newly generated spines and adjacent persistent spines. Data are presented as the mean ± SEM, (neurons at 16–21 DIV, n = 15 spines from 3 independent time-lapse sessions for control [KN92], n = 14 spines from 3 independent time-lapse sessions for KN93), **p < 0.01, t-test.

Our immunofluorescence data for active Rap1 indicated that Rap1 is activated postsynaptically. To gain insights into the spatio-temporal dynamics of Rap1 activation in live neurons, we visualized local Rap1 activity using a fluorescent resonance energy transfer (FRET)-based sensor of Rap1^27,28^ treated with or without the CaMKII inhibitor KN93 (Fig. 5a,b). Without KN93, frequent local activation of Rap1 was observed in both dendritic spines and shafts. This transient activity was greatly suppressed in neurons treated with KN93 (Fig. 5c). When we compared the FRET signals of newly generated spines and adjacent old spines, we noted that stronger FRET signals were more frequent in new spines than in old spines. However, this trend was eliminated by KN93 treatment (Fig. 5d). Thus, live-cell FRET imaging indicated that Rap1 activity is precisely regulated in new spines, an effect that may be responsible for spine destabilization and elimination.

### Rap1 manipulation affects CaMKII-dependent spine regulation in slice cultures

**Figure 6:**
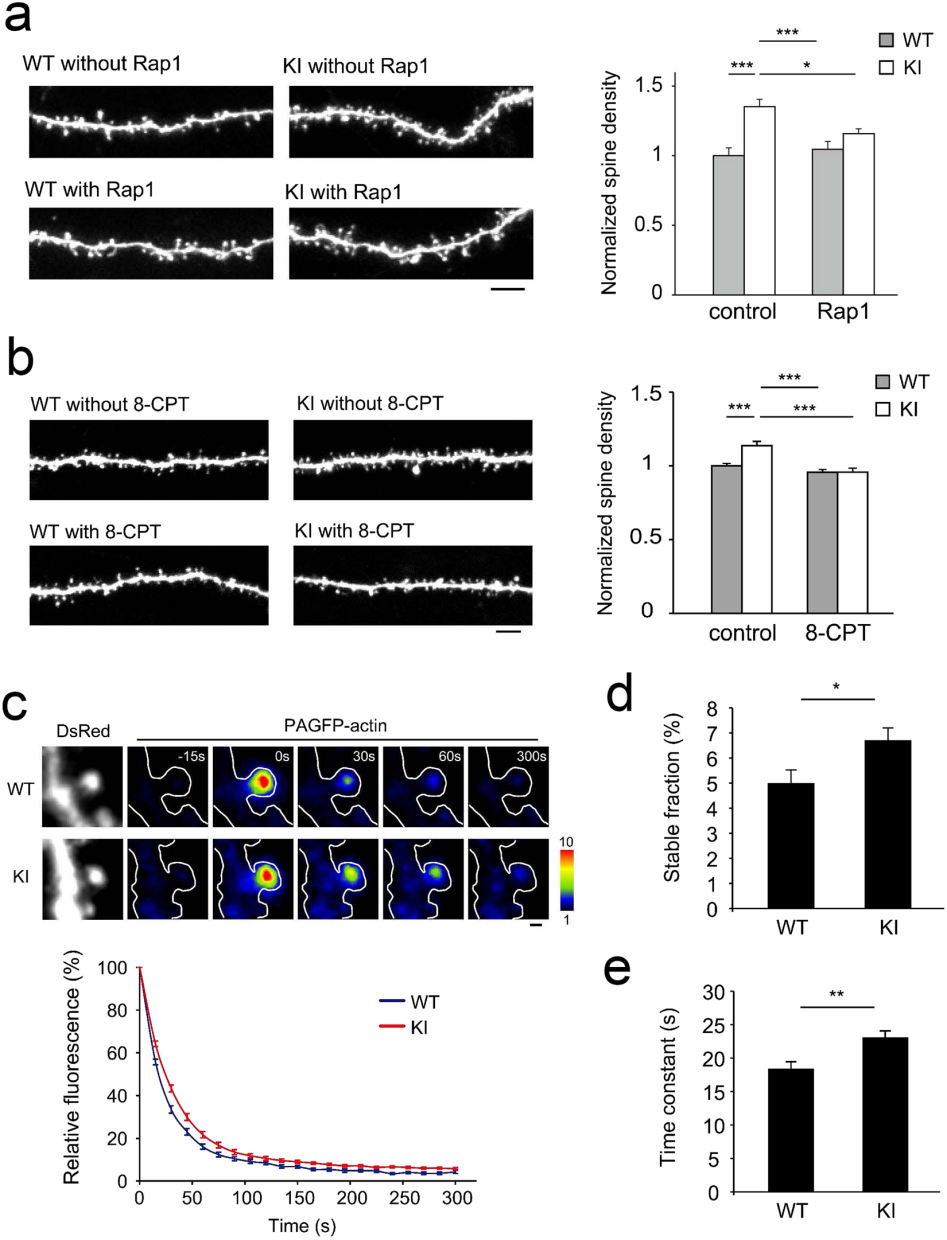
Relationship among CaMKII function, Rap1 activity, and spine actin dynamics. (**a**) Quantification of spine density in Rap1-expressing neurons from wild-type or KI slice cultures. Data are presented as the mean ± SEM, (neurons at 22–25 DIV, n = 11 cells from 3 independent cultures for wild-type control, n = 16 cells from 3 independent cultures for KI control, n = 15 cells from 3 independent cultures for wild-type overexpressing Rap1, n = 25 cells from 3 independent cultures for KI overexpressing Rap1). Bar, 5 μm. ***p < 0.001, t-test. (**b**) Quantification of spine density in wild-type or KI neurons from slice cultures treated with 8CPT-2Me-cAMP (50 μM). Data are presented as the mean ± SEM, (neurons at 21–23 DIV, n = 15 cells from 3 independent cultures for wild-type control, n = 15 cells from 2 independent cultures for KI control, n = 14 cells from 2 independent cultures for wild-type with 8CPT-2Me-cAMP, n = 13 cells from 3 independent cultures for KI with 8CPT-2Me-cAMP). Bar, 5 μm. ***p < 0.001, t-test. (**c**) Images of DsRed2 and PAGFP-actin (pseudocolour coded) in the single spines of wild-type or KI neurons (upper images). Fluorescence decay time-course of PAGFP-actin after photoactivation (lower graph). Bar, 1 μm. (**d** and **e**) Quantification of the stable fraction (**d**) and time constant (**e**) of PAGFP-actin in wild-type and KI neurons (neurons at 19–22 DIV, n = 28 spines from 3 independent cultures for wild-type, n = 27 spines from 2 independent cultures for KI). *p < 0.05, **p < 0.01.

To confirm the relationship between CaMKIIα activity and Rap1 activity, we next overexpressed Rap1 in both wild-type and CaMKIIα KI slice-cultured neurons and measured spine density. Rap1 overexpressin induced a reduction in spine density in CaMKIIα KI neurons but did not affect spine density in wild-type neurons (Fig. 6a). Pharmacological activation of Rap1 via 8CPT-2Me-cAMP, an activator of exchange proteins directly activated by cAMP (Epac, a guanine nucleotide exchange factor of Rap1)^29^, showed effects similar to those of Rap1 overexpression, supporting the idea that CaMKIIα-dependent spine regulation is mediated by Rap1 activation (Fig. 6b).

### Stabilization of actin in CaMKIIα KI neurons revealed by imaging of photoactivatable GFP-tagged actin

Actin rearrangements have been associated with the structural remodelling of spines^30,31^, and Rap1 has been postulated to be a regulator of actin reorganization^32^. CaMKIIα KI neurons show reduced Rap1 activity, which may suppress actin dynamics in spines. Decay experiments involving actin tagged with photoactivatable GFP (PAGFP) in single spines have demonstrated the presence of fractions of stable and dynamic pools of actin and the decay time-constants of the dynamic pool (Fig. 6c)^33^. The fraction of stable actin was significantly higher in CaMKIIα KI slice-cultured neurons than in wild-type neurons (Fig. 6d), suggesting that the spines in CaMKIIα KI neurons are more stable than those in wild-type neurons. The decay time-constant was also increased in CaMKIIα KI neurons (Fig. 6e), suggesting that the exchange of F-actin subunits was slower in these neurons than in wild-type neurons. Both the increase in the size of the stable actin pool and the slower exchange of actin polymers are indicative of the stabilization of the actin cytoskeleton in CaMKIIα KI neurons. Fluorescence decay experiments suggest that the increased rate of spine addition in CaMKIIα KI neurons may result from the stabilization of newly formed spines by increases in F-actin stability.

### Regulation of synGAP by CaMKII activity under basal conditions

**Figure 7:**
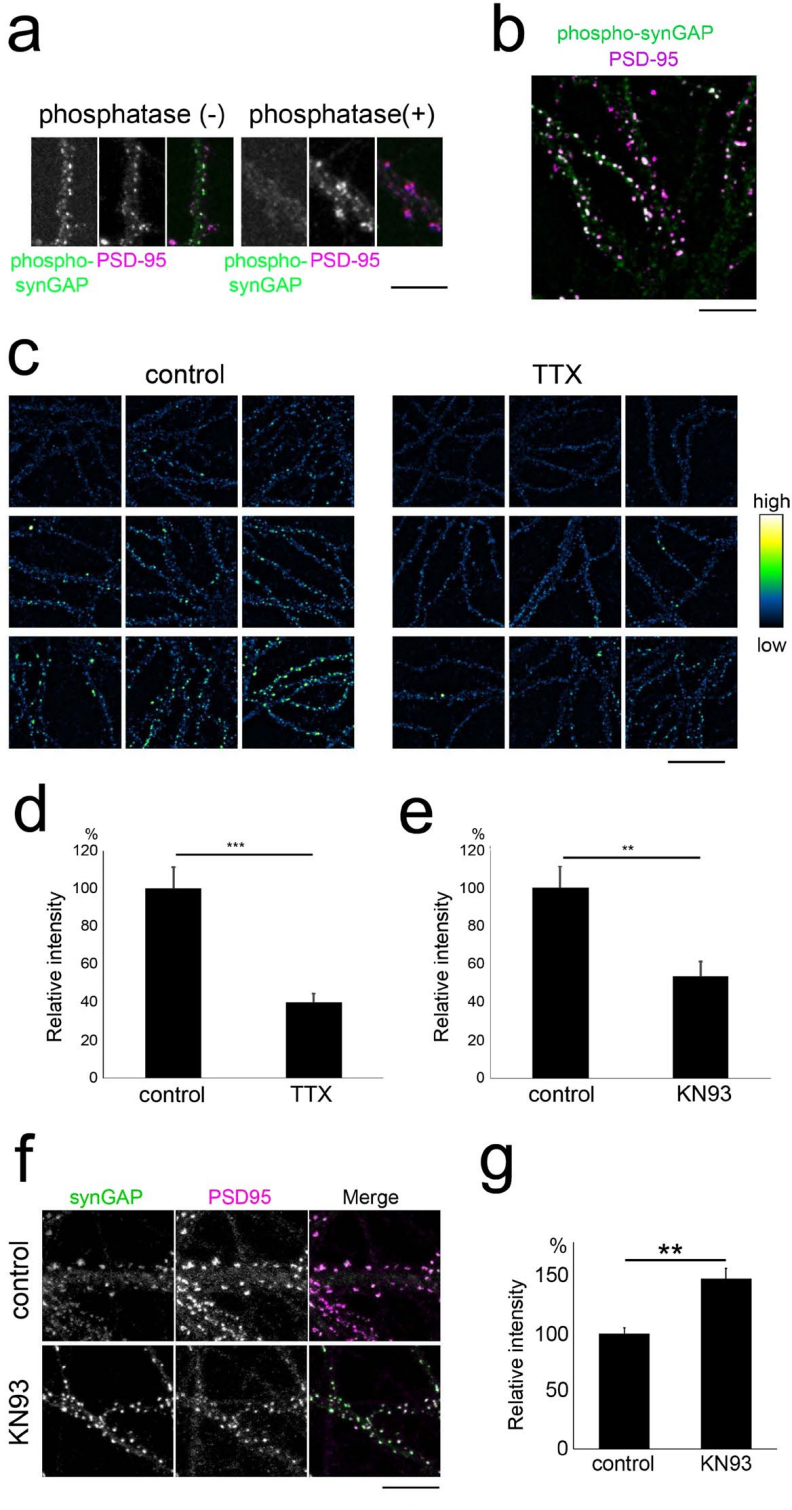
Phosphorylation and dispersion of synGAP by CaMKII. (**a**) The specificity of the anti-phosphorylated synGAP antibody. Phosphatase treatment induced a marked reduction in anti-phosphorylated synGAP immunoreactivity in neurons at 19 DIV. Bar, 10 µm. (**b**) Phosphorylated synGAP molecules are enriched at postsynaptic sites marked by an anti-PSD-95 antibody. Bar, 10 µm. (**c**) TTX treatment reduces phosphorylated synGAP immunoreactivity. Bar, 20 µm. (**d**) Reduction in phosphorylated synGAP immunoreactivity following TTX treatment. Data are presented as the mean ± SEM, (neurons at 16–19 DIV, n = 25 cells from 3 independent cultures for both control and TTX treatment), ***p < 0.001, t-test. (**e**) Reduction in phosphorylated synGAP immunoreactivity following KN93 treatment. Data are presented as the mean ± SEM, (neurons at 16–19 DIV, n = 23 cells from 3 independent cultures for both control (KN92) and KN93 treatment), **p < 0.01, t-test. (**f**) Increase in postsynaptic synGAP clustering following KN93 treatment. Bar, 10 µm. (**g**) Quantification of postsynaptic synGAP cluster intensity in neurons treated with or without KN93. Data are presented as the mean ± SEM, (neurons at 19 DIV, n = 23 cells from 3 independent cultures [KN92 control] and n = 24 cells from 3 independent cultures [KN93]), **p < 0.01, t-test.

A recent study indicated that robust changes in the distribution of synGAP occur in response to CaMKII-dependent phosphorylation during LTP^34,35^. We next tested whether similar regulation of synGAP localization takes place even under basal conditions in dissociated hippocampal neurons. This culture preparation showed calcium transients at a frequency of >0.2 Hz (data not shown), suggesting that the basal electrical activity was present within the neural network. We visualized the distribution of phosphorylated synGAP in neurons using an antibody specific to a phosphorylated serine residue S1123 of synGAP. This phosphorylation site was previously shown to be within a well-conserved CaMKII consensus sequence and can be phosphorylated by CaMKII *in vitro* ^36^. We confirmed the specificity of this antibody by *in situ* dephosphorylation of fixed neurons in culture (Fig. 7a). S1123-phosphorylated synGAP showed a punctate distribution within dendrites and colocalized with PSD-95, suggesting that the proteins are localized at postsynaptic sites (Fig. 7b). Neurons without suppression of endogenous firing activity showed variable levels of S1123-phosphorylation; however, TTX treatment eliminated this variability and greatly reduced the average intensity of the S1123-phosphorylation signal (Fig. 7c,d). Suppression of CaMKII activity by KN93 induced a similar level of suppression of synGAP S1123 phosphorylation (Fig. 7e). Because synGAP phosphorylation by CaMKII triggered by LTP-inducing stimuli was shown to disperse synGAP from postsynaptic sites^34^, we tested whether the blockade of endogenous CaMKII activity under basal conditions would change the synGAP distribution. The application of KN93 induced a higher level of synGAP clustering at the postsynaptic sites labelled by PSD-95 (Fig. 7f and g). We found that the density of the synGAP clusters was not affected by KN93 treatment; however, the size of the clusters increased (Supplementary Fig. 4), suggesting that inhibiting CaMKII basal activity does not initiate the formation of new synGAP complexes at postsynaptic sites. We conclude that synGAP phosphorylation and localization are regulated by CaMKII even in the setting of basal neuronal activity and may contribute to CaMKII-dependent regulation of Rap1.

## Discussion

In this study, we have provided evidence showing that CaMKIIα activity plays an indispensable role in restricting the density of spines on hippocampal pyramidal neurons during postnatal development. This CaMKIIα-dependent regulation is based on activity-driven suppression of increased spine development and is mediated by actin destabilization through the small GTPase Rap1, which may be functionally regulated by synGAP molecules. We propose that the postnatal increase in CaMKIIα expression in the hippocampus terminates the period of rapid increases in spine density by controlling actin stability through the small GTPase Rap1.

LTP in the hippocampus is mediated by the opening of NMDA receptors and the subsequent activation of the CaMKII complex^1^. We have shown previously that the enzymatic activity of CaMKIIα plays an essential role in the electrophysiological and structural changes associated with LTP by creating and analysing a kinase-dead CaMKIIα knock-in mouse strain^19^. In this work, we utilized this mouse strain and critically evaluated the role of CaMKIIα activity in the regulation of spine development and remodelling. In wild type mice, the upregulation of CaMKIIα protein expression was temporally correlated with the attenuation of spine development. In contrast, new spine addition exceeded spine elimination in CaMKIIα KI mice, resulting in the formation of mature pyramidal neurons with many more synaptic connections. Hence, CaMKIIα activity seems to be crucial for the negative control of spine density in the hippocampus. These findings indicate that CaMKIIα activity is not only important for LTP, as previously reported, but is also important for morphological plasticity during development. The contrast in the roles of CaMKIIα in spine development and plasticity regulation is remarkable. Future *in vivo* studies of spine dynamics by two-photon imaging of CaMKIIα KI mice will clarify the role of CaMKIIα activity in intact neural circuits during postnatal development.

The relationship between CaMKII activity and synGAP-dependent signalling in synaptic plasticity is controversial. CaMKII-dependent phosphorylation of synGAP *in vitro* was reported to increase the RasGAP activity of synGAP^37^, suggesting that CaMKII-dependent inhibition of Ras signalling occurs during LTP induction. However, this idea is inconsistent with the well-established model of LTP, in which CaMKII-dependent regulation of synGAP is thought to have positively affect Ras signalling. This conundrum was recently solved by elegant experiments showing that GFP-tagged synGAP is rapidly dispersed from spines during LTP induction^34^. In this study, we confirmed the localized phosphorylation of synGAP at postsynaptic sites in the setting of basal activity and the reduction of this phosphorylation by CaMKII inhibition. The phosphorylation state of synGAP was correlated with its dispersion from postsynaptic sites and may regulate local Rap1 activation. Our FRET imaging data for active Rap1 are consistent with the idea that Rap1 is activated locally within or near postsynaptic spines, where activated CaMKII phosphorylates synGAP and induces its dispersion. We propose that CaMKIIα activity positively regulates Rap1 by regulating the distribution of synGAP, resulting in the destabilization of F-actin and the suppression of increases in spine density.

**Figure 8:**
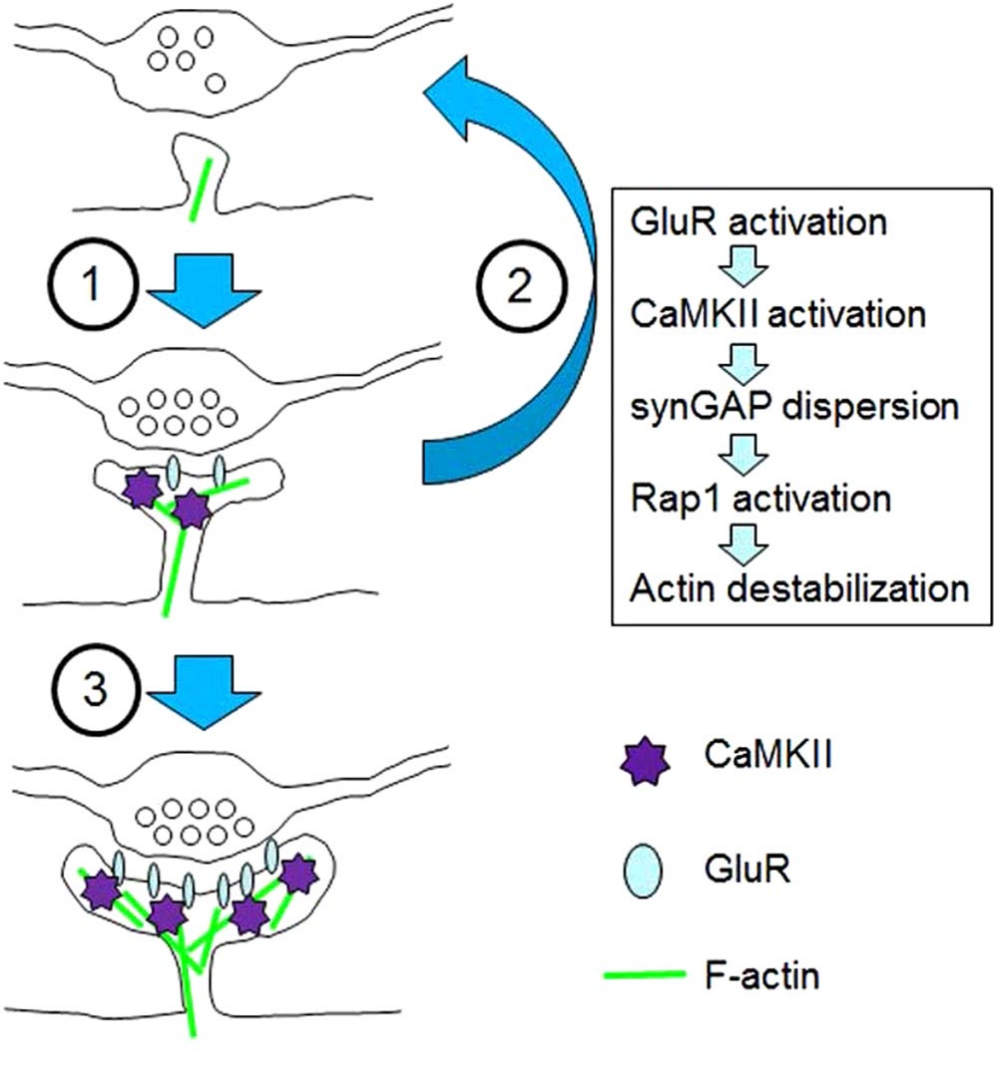
Model for the regulation of spine density by CaMKIIα activity. In the initial stage of spine formation, CaMKIIα has not yet been recruited to spines and Rap1 is not activated. In the subsequent step (arrow 1), glutamate receptors and CaMKIIα molecules begin to accumulate in spines, leading to the phosphorylation of synGAP, the dispersion of synGAP from postsynaptic sites, and the enhancement of local Rap1 activity. Increased Rap1 activity negatively regulates spine formation, possibly by destabilizing F-actin in spines (arrow 2). At a later stage, LTP-like spine-promoting mechanisms begin to operate (arrow 3).

Questions remain regarding how synaptic activity and glutamate receptor activation regulate the CaMKIIα-dependent suppression of increased spine formation. Because the application of glutamate receptor antagonists rescued the spine phenotype in CaMKIIα KI slices, it can be concluded that basal synaptic activity is required for this phenomenon. The involvement of basal synaptic activity and associated CaMKII activation were confirmed by FRET imaging of Rap1 in the presence of the CaMKII antagonist KN93. Although a complex relationship exists among spine remodelling, CaMKIIα, glutamate receptors, and Rap1 GTPase, we propose a model of enhanced elimination of nascent spines by NMDA receptor activation and CaMKIIα (Fig. 8). This activity-dependent pruning does not operate in immature neural circuits, where CaMKIIα expression is low. At a later stage of neural circuit development, CaMKIIα-related signalling begins to operate and to eliminate nascent spines if no additional protective signals are provided. To maintain the appropriate balance between synaptic gain and loss, CaMKIIα-dependent pruning of immature spines may operate in parallel with LTP-like spine protective mechanisms, which are also dependent on CaMKIIα activity^38^. A more thorough quantitative description of the temporal coding of calcium dynamics by the CaMKII complex may explain how multiple CaMKIIα-related signals operate on nascent spines and regulate their fate.

## Methods

### Reagents

The following primary antibodies were used in this study: anti-CaMKIIα (Thermo Scientific), anti-phospho-CaMKIIα (Thr286) (Cell Signaling Technology), anti-synGAP (Thermo Scientific), anti-synGAP (phosphor S1123) (Abcam), anti-active Ras and anti-active Rap1, (NewEast Bioscience), anti-Rap1 (Santa Cruz or Abcam), anti-Ras (Abcam), anti-vGluT1, and anti-PSD95 (Thermo Scientific). The following secondary antibodies were used in this study: Alexa Fluor 546-conjugated anti-rabbit IgG (Invitrogen), Alexa Fluor 488-conjuated anti-mouse IgG (Invitrogen), and horseradish peroxidase (HRP)-conjugated sheep anti-mouse IgG or donkey anti-rabbit IgG ECL (Amersham).

The following addition reagents were used for imaging and pharmacological manipulation: EGFP plasmids (Clontech), the β-actin-promoter EGFP^39^, the β-actin-promoter DsRed2^6^, pCAG-PAGFP-actin^33^, pEGFP-Rap1^24^, a Rap1 FRET probe (Raichu-Rap1)^27,28^, the CaMKII inhibitor KN93 (Sigma-Aldrich), the inactive analogue of KN93 (KN92) (Sigma-Aldrich), (2 R)-amino-5-phosphonovaleric acid (AP-V) (Tocris/Sigma-Aldrich), 6-cyano-7-nitroquinoxaline-2,3-dione (CNQX) (Tocris/Sigma-Aldrich), and the Epac activator 8CPT-2Me-cAMP (Tocris).

The following reagents were used for slice cultures and dissociated neuronal cultures: B27 (Gibco), neurobasal medium (NBM) (Gibco), Eagle’s basal medium (Gibco), Earle’s balanced salt solution (EBSS) (Sigma-Aldrich), heat-inactivated horse serum (Gibco), foetal bovine serum (PAA Laboratories), normal goat serum (PAA Laboratories), glucose (Gibco), HEPES (Gibco), glutamine (CarlRoth), penicillin/streptomycin (PAA Laboratories), Millicell-CM (0.4 µm pore size, Millipore), gold microcarriers, PVP (BioRad), and spermidine (Sigma-Aldrich).

### Dissociated neuronal culture

The animal protocol was approved by the Animal Care and the Use Committee of the University of Tokyo and Fritz Lipmann Institute. Mouse husbandry, anaesthesia, and euthanasia were performed in accordance with the relevant government regulations and institutional guidelines.

The primary cultures of mouse hippocampal neurons were prepared as described previously^40,41^. Hippocampal neurons were grown in minimum essential medium (GIBCO) supplemented with B27 and 5% FCS. The cells were plated on either coverslips or glass-bottom dishes (MatTek) coated with poly-D-lysine. Hippocampal neuron transfection was performed using the calcium phosphate method at 9 days after plating^42^.

### Hippocampal-entorhinal organotypic slices and transfection

Hippocampal slice cultures from P4 or P5 mice were prepared as described previously^43^. The dissected hippocampus and cortex were cut into 400-µm slices with a McIlwain-type tissue chopper. The slices were then placed on a transparent porous filter and incubated at 34 °C in a humidified atmosphere of 5% CO_2_. The slices were treated with culture medium containing 48% Eagle’s basal medium, 24% EBSS, 24% heat-inactivated horse serum supplemented with 5 mg/ml glucose, 10 mM HEPES, and 1 mM glutamine, penicillin, and streptomycin (50 U/ml each). A portion of the entorhinal cortex was left attached to the hippocampal slice. This step prevents the excess sprouting of granule cell axons in the dentate gyrus and thus enables the maintenance of a more physiological synaptic connectivity environment within the cultured hippocampal slice^44^. The slice cultures were transfected at 2 DIV using a biolistic transfection method^45^. Briefly, DNA bullets were prepared using 3.5 mg of gold microcarriers (1 µm in diameter), 35 µl of the β-actin-promoter EGFP (1 mg/ml), 35 µl of spermidine (0.05 M), and 35 µl of CaCl_2_ (1 M). The slices were transfected with a Helios Gene Gun (Bio-Rad) at 80–100 psi. For Rap1 expression, the EGFP-Rap1 fragment was excised from the pEGFP-Rap1 plasmid^24^ and subcloned into an expression vector driven by the β-actin promoter. DNA bullets were prepared with 3.5 mg of gold microcarriers (1 µm in diameter), 17.5 µl of the β-actin-promoter DsRed2 (1 mg/ml), 17.5 of the µl EGFP-Rap1 expression plasmid (1 mg/ml), 35 µl of spermidine (0.05 M), and 35 µl of CaCl_2_ (1 M). The slices were used for structural analyses and time-lapse imaging at 9–31 DIV.

### Pharmacological treatment

Dissociated hippocampal neurons were treated with the CaMK inhibitor KN93 or its inactive analogue, KN92, at a concentration of 10 μM for 5 h before active-Rap1 or phosphorylated synGAP immunocytochemistry (Figs 4 and 7) or for 24 h before spine imaging (Fig. 3). For the spine turnover analysis and FRET experiments involving dissociated neurons, we added KN93 or KN92 to the neurons at 30 min before time-lapse imaging. The dissociated neurons were treated with 1 μM TTX for 2 h before immunocytochemistry for phosphorylated synGAP. The hippocampal slice cultures were treated with 50 μM AP-V or 20 μM CNQX for 4 days for spine density analysis. To activate Rap1, we treated the hippocampal slices with 50 μM 8CPT-2Me-cAMP for 4 days.

### Immunocytochemistry

Neurons were fixed with 4% formaldehyde in PBS for 25 min at room temperature and then washed 3 times with PBS. The cells were subsequently permeabilized with 0.2% Triton X-100 for 5 min at room temperature and blocked with a solution containing 5% normal goat serum in PBS for 1 h. The cells were then incubated with the appropriate primary antibodies in PBS over night at 4 °C, after which they were incubated with the appropriate secondary antibodies in PBS containing 5% normal goat serum for 1 h. For alkaline phosphatase treatment of the fixed neurons, we incubated the samples with 100 µl of alkaline phosphatase solution containing 450 U of bacterial alkaline phosphatase (Invitrogen), 5% β-mercaptoethanol, and 10 mM Tris-HCl (pH 8.0) for 3 h at 32 °C.

### Imaging of dissociated neurons after immunocytochemistry

Images were obtained on a confocal laser-scanning microscope (Fluoview, Olympus) with a 60 × water immersion lens (NA 1.2). Multiple focal planes with z-spacing of 0.4 μm were projected onto a single image using the maximum brightness operation. The fluorescence background was reduced by subtracting the image created by the Gaussian blur operation from the original image. SynGAP, vGluT1, and active Rap1 puncta were automatically selected among the projection images as local increases in fluorescence intensity greater than 0.2 μm^2^. The intensity of the pixels within the area of the synaptic punctae was quantified using ImageJ. Comparisons of the fluorescence intensities were made between samples that were stained simultaneously and imaged with the same acquisition parameters.

### Western blot analysis

Six slices in culture were combined in a single quantitative analysis. The samples were extracted with 70 µl of SDS sample buffer (50 mM Tris-HCl, pH 7.5, 5% (v/v) glucose, 1% (m/v) sodium dodecyl sulfate (SDS), 194 mM bromophenol blue) and boiled for 10 min at 94 °C. The extracts (corresponding to 10 to 30 µg of total protein) were separated by SDS-PAGE, transferred to a Hybond ECL nitrocellulose membrane, washed with PBS and then blocked with 5% skim milk in PBS for 1 h at room temperature. The membranes were then incubated with primary antibodies against CaMKIIα overnight at 4 °C. After being washed and incubated with HRP-conjugated sheep anti-mouse ECL antibodies (Amersham) for 1 h, the signals were visualized using a Perkin Elmer ECL Chemiluminescence Imager. For the purpose of normalization, the membranes were stripped and incubated with antibodies against α-tubulin or β-actin. Several exposure times were used to obtain signals in the linear range.

### Pull-down assay

Wild-type and CaMKIIα KI littermate hippocampi were collected at P28 and homogenized with a Precellys homogenizer at 5000 RPM (2 × 5 s) in 250 µl of ice-cold lysis buffer (the pull-down kit lysis buffer was used, and Roche protease inhibitor complete EDTA-free and phosphatase inhibitors were added to the mixture). The P28 hippocampi corresponded to mature slice cultures that were prepared from P4 mice and maintained *in vitro* for an additional 24 days. We selected this stage of development for protein extraction based on the assumption that the speed of neuronal differentiation is similar between *in vivo* and in slice samples. The nuclei were pelleted at 14,000 × g for 5 min, and the protein concentrations in the supernatant were determined. The supernatant was used for the active GTPase assays, which were performed according to the manufacturer’s protocol. The following amounts of protein were used for the pull-down assays: 650 µg of protein in the active Rap1 pull-down assay (Thermo Scientific) and 700 µg of protein in the active Ras pull-down assay (Thermo Scientific).

### Intracellular injection

For the intracellular injection of Alexa488 dye (Thermo Scientific), we fixed slice cultures with 4% paraformaldehyde for 30–40 min. Micropipettes filled with Alexa488 solution (5–10 mM) were inserted into the cell bodies or thick primary dendrites of CA1 pyramidal neurons, after which a current (0.3–0.4 μA) was applied to the neurons for 3–5 min to deliver the fluorescent dye. The tip resistance of the micropipettes was 100–300 MΩ. The dye-loaded neurons were subsequently imaged, and the spine density was measured for the neurons treated with AP-V and CNQX (Fig. 2).

### Imaging of organotypic slice cultures

Live slice cultures were placed in a chamber containing Tyrode’s solution (in mM: 119 NaCl, 2.5 KCl, 2 CaCl_2_, 2 MgCl_2_, 25 HEPES, 30 glucose, pH 7.4), and the chamber was maintained at 36 °C. Images were obtained on a BioRad Radiance (Figs 1 and 2a–c) or FV1000 confocal laser-scanning microscopes (Figs 2d and 6a–e, Olympus, Tokyo, Japan). The EGFP was excited at 488 nm, and the emitted light was collected with a 500–550-nm band-pass filter. To analyse the spines, we captured a series of high-resolution images (512 × 128) using a 60× water immersion lens (NA 0.9–1.0) and collected images at an additional electric zoom factor of 2.5x–5x. Multiple optical sections (z-spacing of 0.5–0.7 µm) were collected. For each transfected neuron, several segments of apical oblique, apical main, and basal dendrites were imaged and used for spine analysis. The 3D image stacks were projected onto 2D images using an ImageJ maximum-brightness operation. We used the following spine addition and elimination criteria: spines identified on Day 1 that had completely disappeared or whose lengths were reduced (measured as a local increase in the dendrite contour) to <3 pixels (corresponding to 0.15 μm in our imaging setup) were considered lost, and spines that clearly protruded from dendritic shafts at positions at which no spines had been detected on Day 1 and exhibited lengths >3 pixels were considered formed. When the identity of a protrusion was unclear (that is, when it was unclear whether the protrusion was a single spine, two spines, or an axonal profile), the individual z images were re-examined. No effort was made to analyse spines emerging below or above the dendrite, as the resolution of the imaging system was lower in the z direction and insufficient for resolving spines^46^. Imaging of fixed slices with pyramidal neurons that were intracellularly injected with Alexa488 was performed with protocols similar to those described above (Fig. 2d).

### Photoactivation of PAGFP-actin

The expression of both DsRed2 and PAGFP-actin in the same neurons was achieved using a biolistic transfection method at 2 DIV. A mixed solution containing the expression plasmids for DsRed2 and PAGFP-actin was used to prepare DNA bullets. At 19–21 DIV, time-lapse images were obtained with a FV1000 confocal laser-scanning microscope (Olympu, Tokyo, Japan). A 60 × water immersion lens (NA 1.0) was used to acquire these images. PAGFP-actin and DsRed2 were excited by 473 and 559 nm lasers and detected by barrier filters of 490–540 and 575–675 nm, respectively. PAGFP-actin was activated by a 405-nm diode laser (~0.3 mW, 200-ms duration), and fluorescence decay was imaged every 15 s for 5 min. For data analysis, the regions of the spine heads were manually defined based on the DsRed2 signals. After subtraction of the background signals, the total fluorescence of PAGFP-actin on the spine head was measured at each frame. The stable fraction and time constant of PAGFP-actin were calculated by fitting the fluorescence decay to the following first order exponential decay equation using Origin 9.0 (Origin Lab):

$${\rm{y}}={{\rm{y}}}_{0}+A{e}^{-\frac{t}{\tau }}$$

where y_0_ is the stable fraction, and τ is the time constant.

### FRET imaging of active Rap1

Live cells were maintained at 37 °C in a chamber with a continuous flow of humidified 5% CO_2_ to maintain the pH of the medium. Images were acquired using a confocal laser-scanning microscope (Fluoview, Olympus) with 60× or 100× oil immersion lenses (NA 1.1 or NA 1.3). The cells were excited by a 405 nm diode laser, and the fluorescence was detected by barrier filters of 465–495 nm (for CFP fluorescence) and 535–565 nm (for YFP fluorescence). The ratio images were created after subtraction of the fluorescence background value, as estimated from the fluorescence intensity of an imaged area with no discernible fluorescence. For presentation, we created a mask corresponding to the dendritic shape by automatic thresholding of CFP fluorescence. The ratio image was multiplied by the binary mask to produce the masked FRET image^47^. New and persistent spines were classified by their life-time during FRET imaging sessions. Spines that appeared during 4-h imaging sessions and lasted for more than one image frame were classified as new spines, whereas spines that were present throughout the 4-h imaging sessions were classified as persistent spines. The data shown in Fig. 5c are from persistent spines, and the data shown in Fig. 5d compare the FRET signals in new spines at the first time point after their appearance with those in adjacent persistent spines.

### Statistics

Data are presented as the mean ± S.E.M. Normal distribution was tested with the Shapiro-Wilk normality test. Statistical evaluation of the data was performed by two-tailed unpaired t-tests, and two-way ANOVAs. Tukey’s post-hoc tests were applied as indicated in the figure legend. P values < 0.05 were considered statistically significant (*p < 0.05, **p < 0.01, ***P < 0.001). Statistical calculations were performed using R software (R Core Team (2013). R: A language and environment for statistical computing. R Foundation for Statistical Computing, Vienna, Austria, http://www.R-project.org/.) using the Phia package, ii) GraphPad InStat 3.0 or iii) GraphPadPrism 4 (GraphPad Software Inc.).

## Electronic supplementary material

Supplementary Information

## References

[1] Lisman J, Schulman H, Cline H (2002). The molecular basis of CaMKII function in synaptic and behavioral memory. Nat. Rev. Neurosci..

[2] Fink CC (2003). Selective regulation of neurite extension and synapse formation by the beta but not the alpha isoform of CaMKII. Neuron.

[3] Thiagarajan TC, Piedras-Renteria ES, Tsien R (2002). W. alpha- and betaCaMKII. Inverse regulation by neuronal activity and opposing effects on synaptic strength. Neuron.

[4] De Simoni A, Griesinger CB, Edwards FA (2003). Development of rat CA1 neurones in acute versus organotypic slices: role of experience in synaptic morphology and activity. J. Physiol..

[5] Miller M (1981). Maturation of rat visual cortex. I. A quantitative study of Golgi-impregnated pyramidal neurons. J. Neurocytol..

[6] Isshiki M (2014). Enhanced synapse remodelling as a common phenotype in mouse models of autism. Nat. Commun..

[7] Zuo Y, Lin A, Chang P, Gan WB (2005). Development of long-term dendritic spine stability in diverse regions of cerebral cortex. Neuron.

[8] Holtmaat AJGD (2005). Transient and persistent dendritic spines in the neocortex *in vivo*. Neuron.

[9] Lai KO, Ip NY (2013). Structural plasticity of dendritic spines: The underlying mechanisms and its dysregulation in brain disorders. Biochimica et Biophysica Acta - Molecular Basis of Disease.

[10] Alvarez VA, Ridenour DA, Sabatini BL (2007). Distinct structural and ionotropic roles of NMDA receptors in controlling spine and synapse stability. J Neurosci.

[11] Iwasato T (2000). Cortex-restricted disruption of NMDAR1 impairs neuronal patterns in the barrel cortex. Nature.

[12] Herring BE, Nicoll R (2016). Long-term potentiation: from CaMKII to AMPA receptor trafficking. Annu. Rev. Physiol..

[13] Hell JW (2014). CaMKII: Claiming center stage in postsynaptic function and organization. Neuron.

[14] Kim K (2015). A Temporary gating of actin remodeling during synaptic plasticity consists of the interplay between the kinase and structural functions of CaMKII. Neuron.

[15] Matsuzaki M, Honkura N, Ellis-Davies GCR, Kasai H (2004). Structural basis of long-term potentiation in single dendritic spines. Nature.

[16] Komiyama NH (2002). SynGAP regulates ERK/MAPK signaling, synaptic plasticity, and learning in the complex with postsynaptic density 95 and NMDA receptor. J. Neurosci..

[17] Kim JH, Liao D, Lau LF, Huganir RL (1998). SynGAP: A synaptic RasGAP that associates with the PSD-95/SAP90 protein family. Neuron.

[18] Chen HJ, Rojas-Soto M, Oguni A, Kennedy MB (1998). A synaptic Ras-GTPase activating protein (p135 SynGAP) inhibited by CaM kinase II. Neuron.

[19] Yamagata Y (2009). Kinase-dead knock-in mouse reveals an essential role of kinase activity of Ca2+/calmodulin-dependent protein kinase IIalpha in dendritic spine enlargement, long-term potentiation, and learning. J. Neurosci..

[20] Fiala JC, Feinberg M, Popov V, Harris KM (1998). Synaptogenesis via dendritic filopodia in developing hippocampal area CA1. J Neurosci.

[21] Harris KM, Jensen FE, Tsao B (1992). Three-dimensional structure of dendritic spines and synapses in rat hippocampus (CA1) at postnatal day 15 and adult ages: implications for the maturation of synaptic physiology and long-term potentiation. J. Neurosci..

[22] Pokorny J, Yamamoto T (1981). Postnatal ontogenesis of hippocampal CA1 area in rats. II. Development of ultrastructure in stratum lacunosum and moleculare. Brain Res. Bull..

[23] Zhu JJ, Qin Y, Zhao M, Van Aelst L, Malinow R (2002). Ras and Rap control AMPA receptor trafficking during synaptic plasticity. Cell.

[24] Xie Z, Huganir RL, Penzes P (2005). Activity-dependent dendritic spine structural plasticity is regulated by small GTPase Rap1 and its target AF-6. Neuron.

[25] Cahill ME (2016). Bidirectional synaptic structural plasticity after chronic cocaine administration occurs through Rap1 small GTPase signaling. Neuron.

[26] Fu Z (2007). Differential roles of Rap1 and Rap2 small GTPases in neurite retraction and synapse elimination in hippocampal spiny neurons. J. Neurochem..

[27] Mochizuki N (2001). Spatio-temporal images of growth-factor-induced activation of Ras and Rap1. Nature.

[28] Ohba Y, Kurokawa K, Matsuda M (2003). Mechanism of the spatio-temporal regulation of Ras and Rap1. EMBO J..

[29] Enserink JM (2002). A novel Epac-specific cAMP analogue demonstrates independent regulation of Rap1 and ERK. Nat. Cell Biol..

[30] Cingolani LA, Goda Y (2008). Actin in action: the interplay between the actin cytoskeleton and synaptic efficacy. Nat. Rev. Neurosci..

[31] Hotulainen P, Hoogenraad CC (2010). Actin in dendritic spines: Connecting dynamics to function. Journal of Cell Biology.

[32] Liu A (2016). Neuroligin 1 regulates spines and synaptic plasticity via LIMK1/cofilin-mediated actin reorganization. J. Cell Biol..

[33] Honkura N, Matsuzaki M, Noguchi J, Ellis-Davies GCR, Kasai H (2008). The subspine organization of actin fibers regulates the structure and plasticity of dendritic spines. Neuron.

[34] Araki Y, Zeng M, Zhang M, Huganir RL (2015). Rapid dispersion of synGAP from synaptic spines triggers AMPA receptor insertion and spine enlargement during LTP. Neuron.

[35] Yang Y, Tao-Cheng JH, Bayer KU, Reese TS, Dosemeci A (2013). Camkii-mediated phosphorylation regulates distributions of syngap-α1 and -α2 at the postsynaptic density. PLoS One.

[36] Walkup WG (2015). Phosphorylation of synaptic GTPase-activating protein (synGAP) by Ca2+/Calmodulin-dependent protein kinase II (CaMKII) and cyclin-dependent kinase 5 (CDK5) alters the ratio of its GAP activity toward ras and rap GTPases. J. Biol. Chem..

[37] Carlisle HJ, Kennedy MB (2005). Spine architecture and synaptic plasticity. Trends in Neurosciences.

[38] Hill TC, Zito K (2013). LTP-Induced Long-Term Stabilization of Individual Nascent Dendritic Spines. J. Neurosci..

[39] Ebihara T, Kawabata I, Usui S, Sobue K, Okabe S (2003). Synchronized formation and remodeling of postsynaptic densities: long-term visualization of hippocampal neurons expressing postsynaptic density proteins tagged with green fluorescent protein. J. Neurosci..

[40] Okabe S, Miwa A, Okado H (2001). Spine formation and correlated assembly of presynaptic and postsynaptic molecules. J. Neurosci..

[41] Okabe S, Kim HD, Miwa A, Kuriu T, Okado H (1999). Continual remodeling of postsynaptic density and its regulation by synaptic activity. Nat. Neurosci..

[42] Jiang M, Chen G (2006). High Ca2+−phosphate transfection efficiency in low-density neuronal cultures. Nat. Protoc..

[43] Stoppini L, Buchs PA, Muller D (1991). A simple method for organotypic cultures of nervous tissue. J. Neurosci. Methods.

[44] Coltman BW, Earley EM, Shahar A, Dudek FE, Ide CF (1995). Factors influencing mossy fiber collateral sprouting in organotypic slice cultures of neonatal mouse hippocampus. J. Comp. Neurol..

[45] O’Brien JA, Lummis SCR (2006). Biolistic transfection of neuronal cultures using a hand-held gene gun. Nat. Protoc..

[46] Nimchinsky E, Oberlander M, Svoboda K (2001). Abnormal development of dendritic spines in FMR1 knock-out mice. J. Neurosci..

[47] Spiering D, Bravo-Cordero JJ, Moshfegh Y, Miskolci V, Hodgson L (2013). Quantitative ratiometric imaging of FRET-biosensors in living cells. Methods Cell Biol..

