# Supplementary material for: Developmental stage-dependent regulation of spine formation by calcium-calmodulin-dependent protein kinase IIα and Rap1
Source: Sci Rep. 2017 Oct 17;7:13409. doi: 10.1038/s41598-017-13728-y (PMC5645322; doi:10.1038/s41598-017-13728-y)
Supplement: Supplementary file 1 — Supplementary Information [file 41598_2017_13728_MOESM1_ESM.pdf]

Supplementary information

Title: Developmental stage-dependent regulation of spine formation by calcium-calmodulin dependent protein kinase IIα and Rap1

Author list: Solveigh Cornelia Koeberle, Shinji Tanaka, Toshihiko Kuriu, Hirohide Iwasaki, Andreas Koeberle, Alexander Schulz, Dario-Lucas Helbing, Yoko Yamagata, Helen Morrison, and Shigeo Okabe

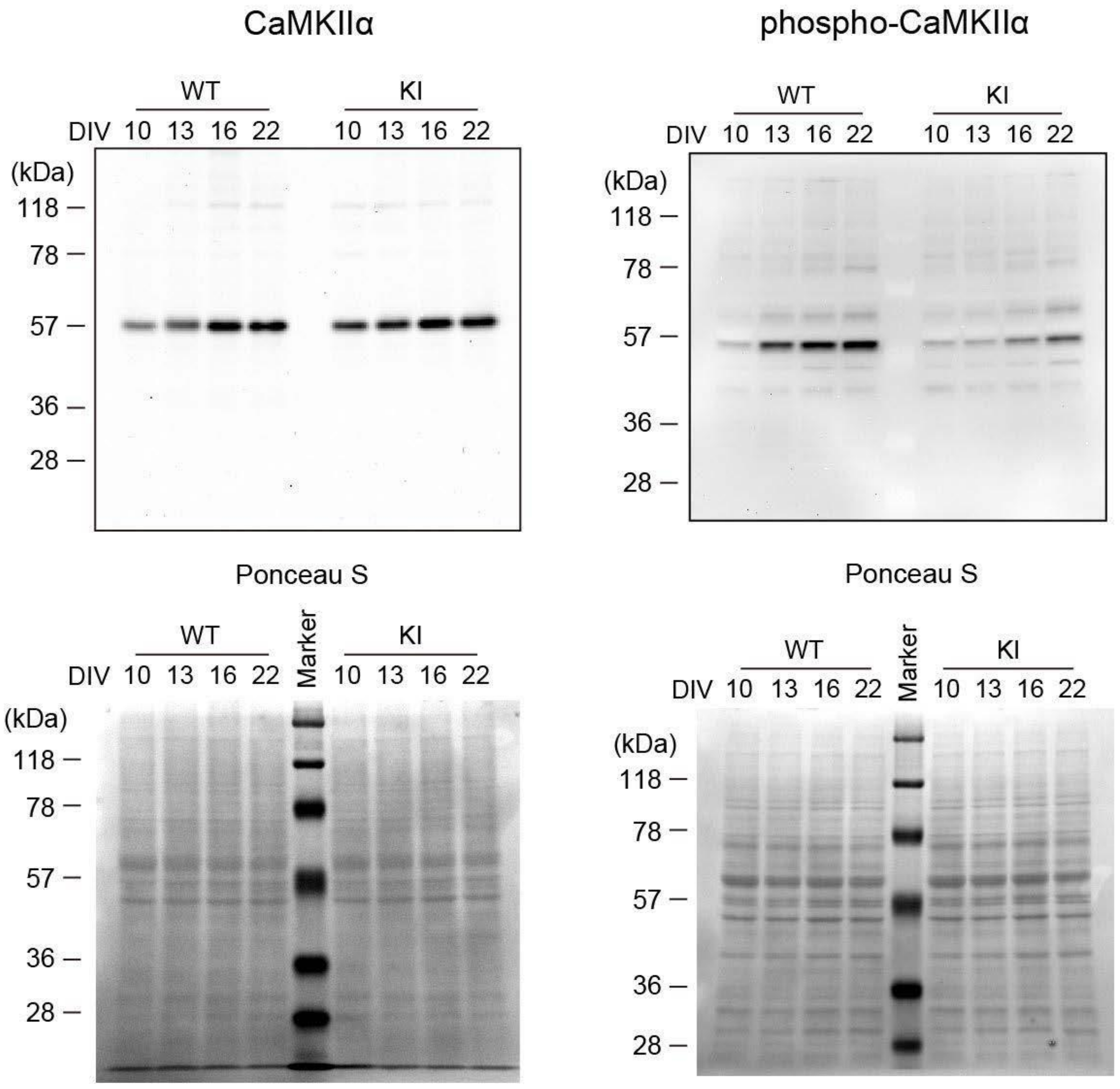

Supplementary Figure 1. Characterization of CaMKIIα protein content and its phosphorylation

Total CaMKIIα protein content and Thr286 phosphorylation in the hippocampal slice culture at DIV10, 13, 16 and 21 or 22 in both wild-type (WT) and CaMKIIα KI (KI). Total CaMKIIα protein content and its Thr286 phosphorylation increase from DIV10 to 16 and stays constant thereafter, irrespective of the genotypes. Upper images show the western blot images of total CaMKIIα and its phosphorylation. A portion of upper left image corresponding to the molecular weight of CaMKIIα (54 kDa) was shown in Figure 1e. Ponceau S staining (lower image) of the blotting membrane confirmed similar amount of protein loading to each well and the molecular weights of marker proteins (118, 78, 57, 36 and 28 kDa).

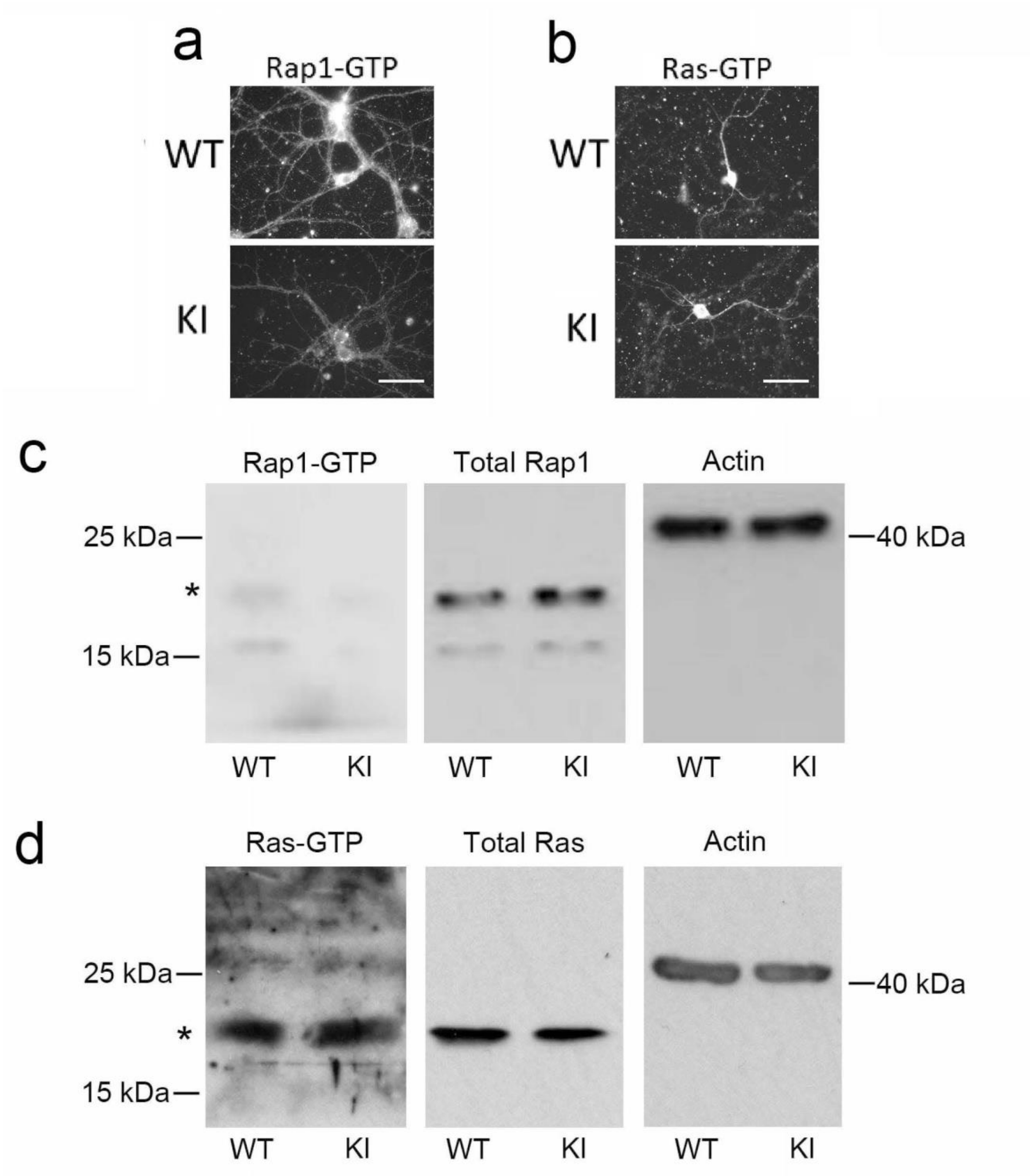

Supplementary Figure 2. Elimination of CaMKIIα activity reduces Rap1 activity.

(a and b) Immunofluorescence of wild-type (WT) or CaMKIIα KI (KI) hippocampal neurons with antibodies against GTP-bound conformation of Rap1 (Rap1-GTP) and Ras (Ras-GTP). Bars, 50 μm.

(c and d) Immunoblotting of pull-down products from hippocampal tissue extracts (P28) of WT and KI mice. Pull-down products and tissue extracts were analysed by antibodies against Rap1 (c) and Ras (d), together with control anti-actin antibody. Level of active Rap1 was reduced whereas level of active Ras was similar. Asterisks indicate the molecular size of Rap1 or Ras.

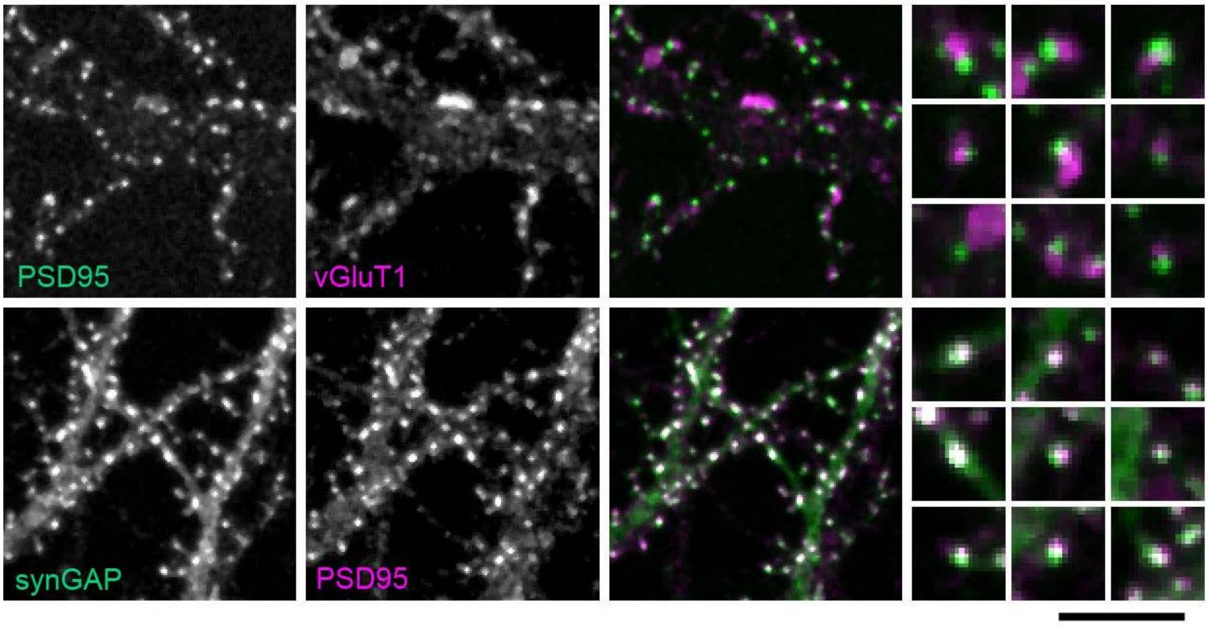

Supplementary Figure 3. Fluorescence images of synapses stained with a presynaptic marker (vGluT1) and postsynaptic markers (PSD95 and synGAP).

Overlap of PSD95-positive postsynaptic structure and vGluT1-positive presynaptic structure is minimal, while synGAP and PSD95 immunoreactivity shows extensive overlap. Bar, 10 and 4 µm for lower magnification images and higher magnification images.

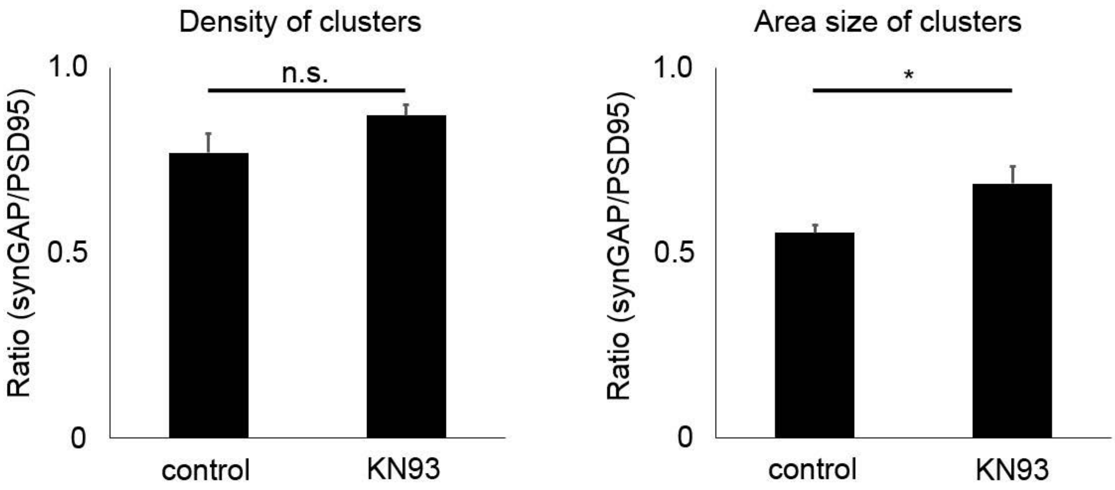

Supplementary Figure 4. Quantification of the density of synGAP clusters and their size.

From the fluorescence images of neurons stained with synGAP and PSD95 antibodies, densities and sizes of synGAP and PSD95 clusters were measured. Neurons were treated with either KN92 (control) or KN93. Density and size of synGAP clusters were normalized against those of PSD95. Data are presented as mean ± SEM, (neurons at 19 DIV, n = 9 cells from 3 independent cultures) n.s. $p > 0.05$, *$p < 0.05$, t-test.
